# The Bioactive Profile, Nutritional Value, Health Benefits and Agronomic Requirements of Cherry Silverberry (*Elaeagnus multiflora* Thunb.): A Review

**DOI:** 10.3390/molecules27092719

**Published:** 2022-04-23

**Authors:** Anna Bieniek, Sabina Lachowicz-Wiśniewska, Justyna Bojarska

**Affiliations:** 1 Department of Agroecosystems and Horticulture, Faculty of Agriculture and Forestry, University of Warmia and Mazury in Olsztyn, Prawocheńskiego 21 Street, 10-720 Olsztyn, Poland; anna.bieniek@uwm.edu.pl; 2 Department of Food and Nutrition, Calisia University, Nowy Świat 4 Street, 62-800 Kalisz, Poland; 3 Department of Horticulture, West Pomeranian University of Technology Szczecin, Słowackiego 17 Street, 71-434 Szczecin, Poland; 4 Chair of Food Plant Chemistry and Processing, Faculty of Food Sciences, University of Warmia and Mazury in Olsztyn, Cieszyński Sq. 1 Street, 10-726 Olsztyn, Poland; justyna.bojarska@uwm.edu.pl

**Keywords:** cherry elaeagnus, chemical composition, biologically active compounds, antioxidant activity, cultivation

## Abstract

The cherry silverberry (*Elaeagnus multiflora* Thunb.) is a lesser-known plant species with high nutritional and therapeutic potential. Cherry silverberry contains numerous biologically active compounds. The cherry silverberry is a shrub growing up to 3 m. Its drupe-like fruit is ellipsoidal, up to 1 cm long, and set on stems. It is red in color, juicy, and sour, and its taste resembles that of red currants. According to the literature, cherry silverberry fruit contains carbohydrates, organic acids, and amino acids, as well as vitamin C, in addition to biominerals, polyphenols, flavonoids, carotenoids, chlorophylls, and tocopherols, which contribute to its high nutritional value. New biotypes of cherry silverberry cultivated in Poland can be used for the production of functional foods and direct consumption. In China, the cherry silverberry, known as goumi, has been used as a medicinal plant and a natural remedy for cough, diarrhea, itch, foul sores, and, even, cancer. This review article summarizes the scant research findings on the nutritional and therapeutic benefits of cherry silverberry.

## 1. Introduction

Bioactive compounds are widespread in the vegetal world. They exert protective effects on plants, as well as human and animal health. Bioactive substances can act as natural antioxidants, whose presence in the body may help prevent a wide variety of lifestyle diseases [1]. Plant species that are rich sources of bioactive substances have been extensively researched around the world [2]. Particular attention has been paid to lesser-known plant species such as kiwiberry, cornelian cherry, honeysuckle, hawthorn, chokeberry, rowanberry, elderberry, medlar, bilberry, seabuckthorn, and silverberry, which grow in different climatic zones and have been introduced to cultivation outside their natural geographic ranges. Novel fruits and berries are increasingly being introduced into local and global food systems [3,4]. Some of them can be eaten raw, while others require processing [5,6]. Neglected and underutilized edible plant species can also boost the livelihoods of small-scale farmers and local producers [7]. This group of plants includes *Elaeagnus multiflora* Thunb. (*Elaeagnaceae*), also known as cherry silverberry, cherry elaeagnus, and goumi. The cherry silverberry belongs to the genus *Elaeagnus* L. and the family *Elaeagnaceae* Juss., which also includes the more popular common seabuckthorn (*Hippophaë rhamnoides* L.) [8,9,10,11]. According to the literature [12,13,14,15,16,17,18,19], *E. multiflora* fruit, which is suitable for direct consumption and processing, can be classified as a “superfood” due to its high content of carotenoids, exogenous amino acids, macronutrients, micronutrients, unsaturated fatty acids, and vitamin C. Fresh and processed silverberries are a valuable source of lycopene, the most potent antioxidant among common carotenoids, which is renowned for its anticarcinogenic effects [18,19,20,21]. The cherry silverberry is native to China, Korea, and Japan [9]. In traditional Chinese medicine, the species is known as a phytosterol-rich plant [20,21,22,23,24]. The fruit, leaves, and young branches of *E. multiflora* can be used as phenolic antioxidant additives and dietary supplements [2,8,22,25,26,27,28,29,30,31,32,33,34,35,36,37,38,39] as well as natural remedies for cough, diarrhea, gastrointestinal disorders, itch, cancer, and bone diseases [8,12,19]. Cherry silverberry seeds are used in dietary therapy and as a functional food for cancer prevention [22,28]. According to Kim et al. [29], *E. multiflora* fruit extract can be applied as a whitening functional cosmetic material, due to the suppression of melanin biosynthesis. Cherry silverberries can be processed at home to prepare juice, compote, jam and jelly, herbal tea, wine, soup, sauces, desserts, candies, pudding, ice-cream topping, fruit leather, and other food products [2,9,11]. Today, this species is grown not only in China but also in the eastern United States and in Europe, including Poland [8,21]. As demonstrated by Bieniek et al. [9], the cherry silverberry thrives in the temperate climate of Poland, as it is easy to cultivate and resistant to diseases.

**Figure 1:**
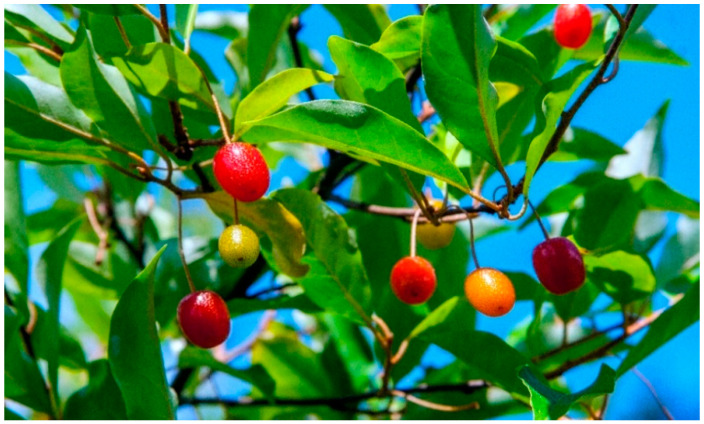
*Elaeagnus multiflora* Thunb. with fruit.

**Figure 2:**
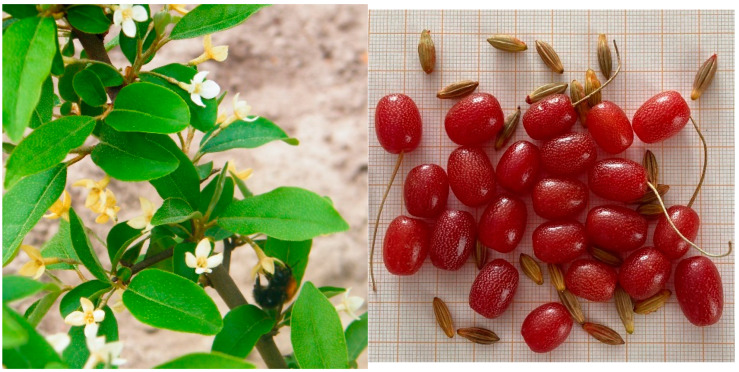
Flowers and fruit with seeds of *Elaeagnus multiflora* Thunb.

*Elaeagnus multiflora* is a thorny shrub, growing up to 3 m (Figure 1). The leaves are typical of the genus *Elaeagnus*—the upper part of the leaf blade is green, whereas its bottom is silvery. Figure 2 presents the flowers and fruit with seeds of *E. multiflora*. The flowers are solitary or in pairs in the leaf axils, fragrant, with a four-lobed pale-yellowish-white corolla 1.5 cm long; flowering occurs in mid-spring. Since silverberry flowers give off a strong aroma, resembling that of cinnamon and vanilla, this plant can be used for flavoring cakes and other desserts [9]. Its drupe-like fruit is ellipsoidal, up to 1 cm long, and set on stems. It is red in color, juicy, and sour, while its taste resembles that of red currants. In Poland, silverberries ripen at the end of June or at the beginning of July [2]. This species is currently being introduced to Russia and the USA, while it has not yet been commercially produced in Poland. Since the 1990s, research has been carried out at the Department of Horticulture, University of Warmia and Mazury in Olsztyn (formerly: University of Agriculture and Technology), to select the most suitable biotypes for cultivation in Poland [2,8,9,26,35,36,37]. According to Lachowicz et al. [2], the cherry silverberry biotypes grown in north-eastern Poland constitute a highly interesting material and could be an excellent source of functional foods. This species also deserves special attention as a fruit plant for organic cultivation.

The aim of this article was to review the latest research findings regarding the cherry silverberry.

## 2. Selection of Varieties and Cultivation Characteristics

The cherry silverberry has been cultivated as a fruit plant since 1974. The first variety of the cherry silverberry, Sakhalinsky pervyi, was bred in the Far Eastern Research Institute of Agriculture in Russia. In 1999, it was entered into the State Register of Breeding Achievements Approved for Use. Other varieties, including Moneron and Taisa (2002), Krilon (2006), Shikotan (2009), Yuzhnyi (2009), Kunashi (2011), Cunai (2015), and Paramushir (2016) were also registered in Russia (State Register of Breeding Achievements Approved for Use, 2016) [9].

A collection of *E. multiflora* was created at the M.M. Gryshko National Botanical Garden (NBG) of the National Academy of Sciences of Ukraine in Kyiv in 1980–1982. The primary material (seeds from free pollination) was imported from Sakhalin (Sakhalin Scientific Research Institute of Agriculture). At present, the *E. multiflora* collection includes 45 genotypes. Grygorieva et al. [22] analyzed the morphometric parameters of fruit in selected genotypes of cherry silverberry grown in the “Forest-Steppe of Ukraine” geographic plot in the M.M. Gryshko NBG. The results of this preliminary study have contributed to increasing interest in *E. multiflora* cultivation among farmers, which can be followed by the domestication and introduction of this species to the agricultural production system in Ukraine and other countries.

In Poland, research into *E. multiflora* was initiated in 1995 at the Department of Horticulture, University of Agriculture and Technology (presently: University of Warmia and Mazury in Olsztyn), when three-year-old plants were obtained from the Institute for Fruit Growing in Samokhvalovitchy in Belarus. [9]. At present, experiments involving several dozen seedlings are being carried out to select the optimal biotypes that could be grown in Poland and other countries [2,8,35,36,37]. Lachowicz et al. [8] noted considerable differences in the chemical composition and antioxidant activity of the *E. multiflora* varieties and biotypes selected at the University of Warmia and Mazury in Olsztyn. Lachowicz et al. [36] found that the fruit of biotypes Si1 and Si2 contained high concentrations of vitamin C, linoleic acid, and α-linolenic acid. The fruit of biotypes Si5 and Si4 was characterized by the highest content of glucose, fructose, and ash, whereas the fruit of biotypes Si0 and Si3 contained the highest levels of the remaining fatty acids as palmitic, oleic, stearic, and organic acids, exhibiting the highest antioxidant activity. Moreover, biotype Si0 had a high content of total polyphenolics, organic acids, and palmitoleic acid, and demonstrated higher antioxidant activity than the remaining biotypes. The above authors concluded that new biotypes of cherry silverberry grown in north-eastern Poland are highly promising and can be consumed raw or used in the production of functional foods.

*Elaeagnus multiflora* varieties ‘Sweet Scarlet’ and ‘SSP’ (seedlings obtained from Austria) can be purchased from Polish nurseries. ‘Sweet Scarlet’ is the earliest-maturing variety. The fruit begins to ripen in the first half of June; the berries remain on the stems for four weeks and, then, fall down. This variety has darker and sweeter fruit than other varieties. ‘Sweet Scarlet’ is an allogamous variety, which requires pollen from another variety for fruit setting. ‘SSP’ is an autogamous variety, with a slower growth rate than ‘Sweet Scarlet’. The fruit ripens at the beginning of July, and it has a sweet taste. Another *E. multiflora* variety is ‘Jahidka’, which produces much shorter shrubs (up to 1.5 m) and red oval fruit weighing 1–1.5 g that ripens in early July [31].

*Elaeagnus multiflora* is often confused with *E. umbellata* because both species have similar leaves and flowers. However, *E. umbellata* produces round fruit with short petioles, typically ripening in September [35].

### Cultivation of Elaeagnus multiflora

The cherry silverberry has low nutritional requirements, and it thrives on dry, sandy, and poor soils. However, the species requires large amounts of sunlight. Cherry silverberry shrubs can grow in the same site for 25 years [9,40]. A symbiosis with nitrogen-fixing actinomycetes makes the cherry silverberry a pioneer soil-fertilizing species [9,40].

**Figure 3:**
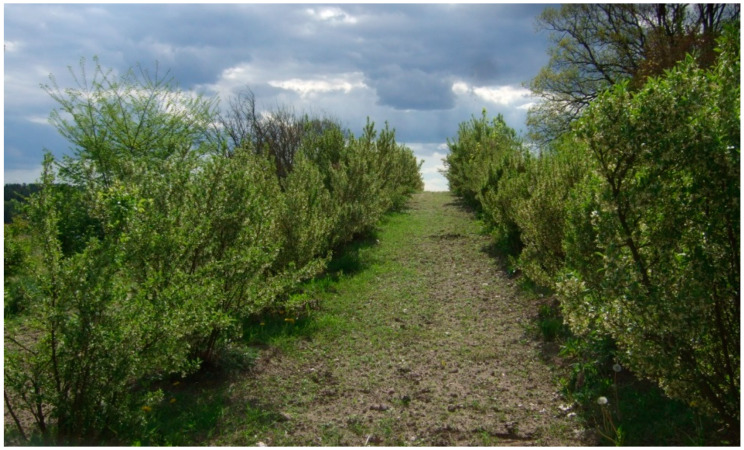
*Elaeagnus multiflora* growing in the Experimental Garden of the University of Warmia and Mazury in Olsztyn (north-eastern Poland).

In commercial plantations, cherry silverberry shrubs should be planted at 4 × 2 m spacing, 5–8 cm deeper than in the seed bed (Figure 3). The species has similar fertilizer requirements to currants and gooseberries. *Elaeagnus multiflora* is highly resistant to drought. Due to its high-quality fruit, it is a promising fruit plant that can be recommended for organic cultivation. Most seedlings begin to bear fruit in the fourth year after planting [21,31]. According to Kołbasina [41], 5-year-old plants can yield 3–4 kg fruit per shrub, 10-year-old plants up to 15 kg, and 20-year-old plants up to 30 kg. Cultivation conditions, as well as climatic factors during the growing season, regardless of genetic factors, have a significant effect on the yield and qualitative characteristics of fruit [9,21]. *Elaeagnus multiflora* can be grown on a small scale and cultivated commercially with the use of combine harvesters [26].

## 3. Biologically Active Compounds in *Elaeagnus multiflora* Thunb.

Cherry silverberry fruit is abundant in bioactive components that are responsible for its health-promoting properties [8,9]. These substances can be divided into primary and secondary metabolites. Primary metabolites are a source of nutrients, energy, and structural components in plants with limited bioactive properties, whereas secondary metabolites are metabolic products in plants that deliver a wide range of health-promoting effects. Primary metabolites include, among others, carbohydrates, organic acids, and amino acids. Secondary metabolites include, among others, vitamin C, biominerals, polyphenols, flavonoids, carotenoids, chlorophylls, and tocopherols (Table 1) [42,43].

Sugars, organic acids, and their ratio can affect the sensory and chemical attributes of the food matrix, including sweetness, microbiological stability, total acidity, pH, and overall sensory acceptability [38]. Therefore, the palatability of cherry silverberry fruit, mainly its sweet and sour taste, is determined by the content of sugars and organic acids. The average content of organic acids in the fruit of *E. multiflora* Thunb. biotypes grown in Poland range from 0.78% to 1.20% [9], or 18.48 to 34.11 g/100 g of dry weight (DW) [2,36], which implies that cherry silverberries are abundant in these compounds. A liquid chromatography analysis revealed the presence of seven organic acids in cherry silverberry fruit: malic, quinic, tartaric, oxalic, citric, isocitric, and succinic acid. The predominant organic acids were malic (55–60% of total organic acids), quinic (11–15%), and tartaric (9–18%) acids [2]. Kim et al. [44] identified four organic acids in cherry silverberry fruit and determined their total content at 294.44 mg/100 g of fresh weight (FW). According to Mikulic-Petkovsek et al. [45], citric and malic acids account for 30–95% of all organic acids in berries. Fruits that are low in citric acid include cherry silverberry as well as chokeberry, rowanberry, and eastern shadbush. Five organic acids with a total content of 167.8 g/100 g FW were identified in cherry silverberry leaves. Malic acid was the predominant compound (66% of total organic acids), followed by acetic (13.7%), citric (8.1%), lactic (6.3%), and succinic acid (5.3%) [17].

Another study demonstrated that cherry silverberry fruit contained 1.54–1.96% of monosaccharides and 5.34–6.30% of total sugars on a fresh weight (FW) basis [9]. Total sugar content was determined at 9.77 to 11.50 ° Brix by Hong et al. [46]. An analysis involving the high-pressure liquid chromatography with refractive index detectors (HPLC-RI) method revealed the presence of two sugars, fructose and glucose. Fructose accounted for around 57–59% and glucose for 41–43% of the total sugars in cherry silverberry fruit [2]. Kim et al. [44] identified five free sugars with a total content of 781 mg/100 g FW in cherry silverberry fruit. Fructose and glucose were the predominant sugars, whereas sucrose, maltose, and trehalose were detected in trace amounts [44]. Cherry silverberry leaves were found to contain five sugars: arabinose, fructose, glucose, maltose, and trehalose. Similar to the fruit, the predominant sugar in the leaves was fructose (46.9% of total sugars), followed by arabinose (27.2%) [17]. According to Mikulic-Petkovsek et al. [45], berries contain mainly fructose and glucose, and fructose accounts for up to 75% of the total sugars. However, some exceptions have been noted, such as kiwifruit, where sucrose represents 71.9% of the total sugars [45].

The sugar–acid ratio denotes the relative content of sugars and acids, which are responsible for the taste and aroma of fruit [45]. Sweet-tasting berries are not always rich in sugar, and they may be low in organic acids, mainly malic acid [45,47]. The sugar–acid ratio affects the perception of sweetness [48], and it ranges from 5.25 to 7.40 in cherry silverberry fruit [16]. In a study by Mikulic-Petkovsek et al. [45], white gooseberries and red, black, and white currants were the most acidic fruits with a sugar–acid ratio of around two. The sweetest-tasting fruits were black mulberries, brambles, and goji berries, with a sugar–acid ratio above 12.9 [45].

Vitamin C (ascorbic acid) is yet another bioactive substance that plays a very important role in fruit. Vitamin C has antioxidant, anticarcinogenic, anti-inflammatory, and antisclerotic properties; it lowers blood glucose levels and reduces the risk of cardiovascular diseases [49,50]. Cherry silverberries are abundant in vitamin C, although the content can vary depending on variety, genotype, growing conditions, weather, and ripeness [9]. In the work of Sakamura et al. [24], vitamin C concentration decreased in successive stages of fruit ripening. In contrast, in *Rubus sieboldi*, *Ribis nigrum*, pears, peaches, and papayas, the content of L-ascorbic acid increased with ripening [24]. In a study by Kim et al. [44], cherry silverberries grown in Korea contained 131.35 mg/100 g FW of ascorbic acid and 431.37 mg/100 g FW of dehydroascorbic acid, and the total content of vitamin C was determined at 562.72 mg/100 g FW. These results indicate that cherry silverberry fruit is an excellent source of vitamin C. In a study conducted by Bieniek et al. [9], the concentration of vitamin C in the fruit of cherry silverberry grown in Poland ranged from 4.22 to 7.70 mg/100 g FW. Vitamin C levels reached 15.8–33.1 mg/100 g in cherry silverberry fruit grown in Ukraine [51] and 27.8 mg/100 g in the fruit grown in Pakistan [52]. In other fruit, vitamin C concentrations were 30 mg/100 g in elderberries, 35–90 mg/100 g in blackcurrants, and 16–32 mg/100 mg in raspberries [53].

Cherry silverberries are also abundant in biominerals, mainly potassium (1627.44 mg/100 g FW), magnesium (140.28 mg/100 g FW), sodium (56.70 mg/100 g FW), calcium (14.70 mg/100 g FW), iron (7.98 mg/100 g FW), manganese (5.53 mg/100 g FW), zinc (2.89 mg/100 g FW), copper, lithium, and nickel (0.10–0.20 mg/100 g FW) [44]. According to Polish Standards [54], 100 g of cherry silverberry fruit provide approximately 65% of the recommended daily intake of potassium, 33–43% of magnesium, 53–79% of iron, 26–32% of zinc, and 240% of manganese, for healthy middle-aged adults [54]. Bal et al. [55] found that seabuckthorn is also a rich source of potassium, whose content was determined at 1012–1484 mg/100 g FW in fruit flesh and at 933–1342 mg/100 g FW in seeds. Cherry silverberry leaves can be used as functional food additives [8], and they have been found to contain 14 minerals with a total content of 1353.70 mg/100 g FW [17]. Similar to the fruit, 100 g of cherry silverberry leaves provided 33% of the recommended daily intake of potassium, 36% of calcium, 35–63% of iron, 22% of copper, 18–24% of magnesium, around 250% of manganese, and around 180% of selenium [54]. Other elements, including Li, Na, Al., Fe, Co, Ni, Cu, Zn, and Ge, were detected in trace amounts [17].

Free and bound amino acids and their derivatives are yet another important group of biologically active compounds. According to Kim et al. [44], cherry silverberries are abundant in amino acids, whose total content was determined at 89.68 mg/100 g FW. The content of serine, alanine, phosphoethanolamine, and β-alanine exceeded 10 mg/100 g FW, whereas aspartic acid, cystine, methionine, phosphoserine, threonine, glutamic acid, glycine, valine, isoleucine, leucine, tyrosine, phenylalanine, taurine, sarcosine, α-aminoisobutyric acid, β-aminoisobutiryc acid, and ornithine were detected at concentrations below 5 mg/100 g FW. In turn, cherry silverberry leaves contained 7 essential amino acids, 10 non-essential amino acids, and 11 amino acid derivatives, with a total content of 943 mg/100 g FW. The following amino acids were identified at concentrations higher than 50 mg/100 FW: threonine, valine, isoleucine, leucine, phenylalanine, glutamic acid, alanine, and tyrosine. Lysine, aspartic acid, serine, cystine, histidine, proline, glycine, tyrosine, arginine, phosphoserine, sarcosine, α-aminoadipic acid, β-aminoisobutyric acid, y-aminoisobutyric acid, and anserine were detected at concentrations below 20 mg/100 g FW. Trace amounts of carnosine, β-alanine, cystathionine, and α-aminoisobutyric acid were also identified in cherry silverberry leaves [17]. The content of amino acids was similar in medlar leaves, but it was 10 times higher in ripe medlar fruit [56]. Amino acid concentrations in Saskatoon berries were estimated at 490 mg/100 g [57]. According to Zhang et al. [58], the content of free amino acids in fruits is determined mainly by ripeness, growing conditions, position on a plant, genotype, and the applied analytical methods.

Cherry silverberries are abundant in bioactive components, with antioxidant properties that deliver numerous health benefits, including polyphenols and isoprenoids [8]. These compounds promote a healthy oxidant/antioxidant balance and lower the risk of chronic non-infectious diseases, such as cardiovascular diseases, cancer, neurodegenerative disorders, diabetes, and obesity [59]. The total content of polyphenolic compounds in *E. multiflora* fruit, expressed in gallic acid equivalents (GAE), was determined at 280 mg/100 g FW by Kim et al. [44], at 12.21 mg% by Hong et al. [46], and at 568 mg GAE/100 g DW by Lachowicz et al. [2]. Polyphenol concentrations are similar in seabuckthorn fruit, where they range from 128.66 to 407.48 mg GAE/100 g [60,61]. High-performance liquid chromatography methods have been applied to assess the content and qualitative composition of polyphenols in cherry silverberry fruit [8,13,37]. Total polyphenol content was determined at 904.65–1268.90 mg/100 g DW in the fruit of the ‘Jahidka’ and ‘Sweet Scarlet’ varieties grown in Poland, after extraction with 30% ethanol [8]; 353 mg/100 g FW in Korean-grown fruit, after extraction with 50% ethanol [13]; and 417.02–819.04 mg/100 g DW in the fruit of Polish-grown biotypes, after extraction with 30% ethanol [37]. According to Cho et al. [62], differences in polyphenol concentrations may be attributed to variety, species, growing conditions, extraction methods, analytical methods, technological process, or the analyzed materials. Lee et al. [13] identified 13 polyphenolic compounds that were classified as flavan-3-ols (epigallocatechin, catechin, epicatechin, epigallocatechin gallate, epicatechin gallate, catechin gallate) and phenolic acids (gallic acid, protocatechuic acid, tannic acid, *p*-hydroxybenzoic acid, vanillic acid, *p*-coumaric acid, ferulic acid). Epicatechin gallate was the dominant flavan-3-ol (66%), whereas gallic acid and *p*-coumaric acid accounted for 26% and 23% of the total phenolic acids, respectively [13]. Lachowicz-Wiśniewska et al. [2,37] identified 16 polyphenols in the fruit of the cherry silverberry varieties ‘Jahidka’ and ‘Sweet Scarlet’, including one phenolic acid, one hydrolysable tannin, one stilbene, and 13 flavanols, as well as polymeric procyanidins. Polymeric procyanidins were the predominant compounds that accounted for 66.0–95.0% of total polyphenols, as evidenced by a mildly astringent taste [63]. In *E. umbellata* fruit, flavonols were the predominant polyphenols (78.8%) [64]. In *E. multiflora* fruit, flavonols—quercetin derivatives, kaempferol, and isorhamnetin—accounted for 5% of the total polyphenols, whereas the content of phenolic acids (sinapic acid derivatives) was determined at 0.2%, hydrolyzable tannins (galloyl derivatives) at 0.3%, and stilbenes (glucosylphloretin derivatives) at 0.2%. Kaempferol-pentoside-rutinoside was the predominant flavonol [37]. In turn, cherry silverberry leaves were found to contain 38 polyphenolic compounds, including three phenolic acids, 35 flavonols, and polymeric procyanidins. Polymeric procyanidins were also dominant and accounted for around 81% of the total polyphenols [2].

Isoprenoids, including carotenoids, chlorophylls, and tocopherols, are indirectly responsible for the color, taste, and aroma of fruits. Cherry silverberry fruit contains carotenoids, whereas chlorophylls have been identified in leaves. Carotenoids are highly biologically active compounds that boost immunity and prevent inflammations caused by excessive formation of reactive oxygen species (ROS) [65,66,67,68]. Chlorophylls stimulate intestinal peristalsis, lower blood pressure, and decrease the risk of anemia [65,67]; plants can hardly bear to live without chlorophyll. Lachowicz et al. [2,37] were the first research team to examine the content as well as the qualitative and quantitative composition of carotenoids and chlorophylls in cherry silverberry fruit [2,37]. Isoprenoid concentrations ranged from 95.69 to 170 mg/100 g DW in the fruit of Polish-grown biotypes, and from 40.09 to 97.15 mg/100 g DW in the fruit of the ‘Sweet Scarlet’ and ‘Jahidka’ varieties. The content of carotenoids ranged from 66.20 to 71.26 mg/100 g DW, and the content of chlorophyll ranged from 1634 to 1694 mg/100 g DW in the ‘Sweet Scarlet’ and ‘Jahidka’ varieties, whereas in the analyzed biotypes, carotenoid concentration was determined at 81 mg/100 g DW and chlorophyll concentration at 393 mg/100 g DW [2,37]. The carotenoid content of seabuckthorn fruit, which belongs to the same family as the cherry silverberry, ranged from 10 to 120 mg/100 g FW [69]. Sixteen carotenoid compounds were identified in cherry silverberry fruit, including eight lycopene derivatives, α- and β-carotene (provitamin A), their two derivatives, lutein, two violaxanthins, and neoxanthin [2,37]. Lycopene delivers numerous health benefits [70], and it was the dominant carotenoid (80%) in cherry silverberry fruit. The remaining carotenoids also have health-promoting properties [66,67,71]. Phytoene is a valuable, but rarely identified, carotenoid. This colorless compound is characterized by high dietary bioavailability, and recent research has shown that phytoene exhibits high levels of biological activity and exerts protective effects on the skin [66]. Seabuckthorn berries are more abundant in β-carotene (0.9–18 mg/100 g FW) than fruits and vegetables that are regarded as the richest sources of this compound [72]. Cherry silverberry fruit contains even more β-carotene (37–42 mg/100 g DW) [2]. Significant differences in carotenoid levels in the analyzed fruits could be related to numerous factors, such as climate, genotype, and agrotechnology [66,67,71]. The fruit of the studied cherry silverberry biotypes also contained α-tocopherol at 3.31–7.07 mg/100 mg DW. In turn, the content of α-tocopherol in cherry silverberry seeds ranged from 2.0 to 3.3 mg/100 g DW [37]. α-tocopherol and its derivatives, known as vitamin E, are powerful antioxidants that delay cell aging [41]. In a study by Piłat et al. [72], tocopherol levels in seabuckthorn berries ranged from 3.35 to 6.27 mg/100 g FW, and α-tocopherol was the predominant compound that accounted for 62–67% of the total tocopherols [73].

## 4. Health-Promoting Properties of *Elaeagnus multiflora* Thunb.

For thousands of years, plants have been used to treat various human and animal diseases [74,75]. Plants of the family *Elaeagnaceae* have gained popularity in recent years due to their exceptional chemical composition as well as health benefits. Seabuckthorn (*Hippophaë rhamnoides* L.) is the most researched representative of this family. Its berries contain more than 190 bioactive compounds, and it is considered a wonder of nature. The cherry silverberry is referred to as a “wonder berry” in the Far East [31,35]. Not only the fruit, but also other plant parts such as flowers, leaves, roots, and stems have been utilized in traditional medicine. Scientific studies have confirmed the antioxidant [13,25,76,77,78,79,80], anti-inflammatory [13,30,33,34], antiproliferative [12,32,81], anticancer [12,81,82], antimicrobial [19], antidiabetic [31,37,80] anti-fatigue [83], and alleviating [82] properties of *E. multiflora.*

Jung et al. [83] examined the effect of *Elaeagnus multiflora* fruits (EFM) on fatigue and exercise performance in BALB/c mice. These results suggest that EMF can be utilized as an efficacious natural resource for its anti-fatigue effects. Subsequent studies Jung et al. [83] conducted on aging male rats suggest that *Elaeagnus multiflora* and *Cynanchum wilfordii* can be effectively used to alleviate testosterone deficiency syndrome (TDS).

### 4.1. Antioxidant Activity

High antioxidant activity has become a topic of numerous studies [76]. The consumption of food that is rich in antioxidants reduces the risk of developing chronic diseases and oxidative stress [77,78]. The development of chronic, autoimmune, neurodegenerative, and metabolic diseases, as well as cancer, is positively correlated with oxidative stress [79]. Phenolics, as metabolites, possess antioxidant activity and can protect the body from damage caused by free-radical-induced oxidative stress (ROS) [24]. Oxidative stress, that is, the imbalance of antioxidants and prooxidants in favor of prooxidants, is caused by high levels of reactive ROS. In free radical processes, ROS react with cellular components, which leads to their modification and damage. A study investigating the total phenolics from different parts of *E. multiflora* from Gilgit-Baltistan (Pakistan) [25] revealed that this plant species is a good candidate for a natural antioxidant. It contains nutritional and functional material in its fruit, leaves, and young branches, and is able to repair damage caused to cells by ROS [25]. The results of the cited study indicate that the concentrations of phenolics in medicinal plant species vary across different plant parts and are affected by the nature of solvents. Lee et al. [13] demonstrated that the 50% ethanol extract of *E. multiflora* fruit displayed the highest antioxidant activities in ABTS^•+^ (2,2′-azino-bis(3-ethylbenzothiazoline-6-sulfonic acid) and DPPH^•^ (1,1-diphenyl-2-picrylhydrazyl) radical scavenging and power-reducing assays. The cited authors suggested that this extract may be used as a natural source for food supplements and pharmaceuticals, due to its strong biological activities and high phytochemical content. According to Ismail et al. [25], *E. multiflora* is rich in bioactive phenolic compounds, which should be isolated for further investigations.

According to Lizardo et al. [80], extracts of cherry silverberry fruits fermented by pure cultures of *Lactobacillus plantarum* KCTC 33131 and *L. casei* KCTC 13086 exhibited favorable physicochemical properties and enhanced phytochemical content, antioxidant properties (DPPH radical scavenging activity, reducing power, superoxide dismutase-like property and hydrogen peroxide scavenging activity), and α-glucosidase and tyrosinase enzyme inhibitory activity, as compared with unfermented fruits. Despite a decrease in the specific phenolic acid contents among the fermented samples, the cherry silverberry fruit, fermented by mixed cultures of *Lactobacillus plantarum* and *L. casei*, contained superior total polyphenols and total individual flavonoid contents in comparison with fruits fermented by single cultures and unfermented ones

### 4.2. Antimicrobial Properties of Elaeagnus

Microbes (such as bacteria, fungi, and viruses) are the major causative agents of infectious diseases, which pose threats to public health [74,75]. The search for plants with antimicrobial activity has gained importance in recent years, due to a growing concern about the increasing rates of infections caused by antibiotic-resistant microorganisms [84,85,86,87,88,89]. Several plant-derived products, such as essential oils and extracts, have been used as traditional antiseptics and have been reported to possess moderate to significant levels of antimicrobial properties. Extracts from plants of the genus *Elaeagnus* were found to be more active against Gram-positive than Gram-negative bacteria [74,90,91]. The antimicrobial activities of selected *Elaeagnus* species, namely *E. angustifolia* [92,93,94], *E. macrophylla* [95], *E. mollis* [58,96], *E. kologa* [97], *E. umbellate* [19,98,99], *E. maritime*, *E. submacrophylla* [100], and *E. indica* [74,101], have also been documented. In the work of Ismail et al. [25] and Nikolaeva et al. [102], epigallocatechin from *Elaeagnus galabra* has been recognized as an antibacterial agent. According to Zargari [103], the leaves and fruit of *E. angustifolia and E. multiflora* exhibit antipyretic activity. Bacterial and fungal strains that are inhibited by *E. multiflora* extracts should be analyzed and characterized in more detail. Mubasher et al. [99] studied the antibacterial activity of *E. umbellate*, which is often confused with *E. multiflora* due to similarities in leaf and fruit morphology. The objective of their study was to evaluate the biological activity of *E. umbellata* extracts against standard microbial strains as well as multi-drug-resistant bacteria isolated from hospitals. Flowers, leaves, and berries were extracted in different solvents and were tested for their antibacterial activity by the disc diffusion method on selected organisms, such as the methicillin-resistant *Staphylococcus aureus* (*S. aureus*), multi-drug resistant *Pseudomonas aeruginosa (P. aeruginosa),* and enterohemorrhagic *Escherichia coli (E. coli).* Most of the extracts displayed broad-spectrum activity, since Gram-positive bacteria, including *S. aureus* and *B. subtilis*, as well as Gram-negative bacteria, including *E. coli* and *P. aeruginosa*, were inhibited. Srinivasan et al. [74] demonstrated that the leaf extracts of *E. indica* possess potent antimicrobial activities. They exerted varied inhibitory effects on the tested microbes. Most polar extracts exhibited strong antimicrobial activities [74,101]. The extracts of *E. umbellata* [104] and *E. indica* exerted greater inhibitory effects on bacteria than fungi. According to Piłat and Zadernowski [72], seabuckthorn leaves contain compounds that inhibit the growth of microorganisms such as *Bacillus cereus*, *Pseudomonas aeruginosa*, *Staphylococcus aureus*, and *Enterococcus faecalis* [105]. Moreover, seabuckthorn seed oil exhibits antibacterial activity against *Escherichia coli* [106].

The above findings indicate that *E. multiflora* can be used in the treatment of infectious diseases. The antimicrobial efficacy of various *Elaeganus* species has already been documented, but further research is needed to identify all of their bioactive compounds [19].

### 4.3. Antidiabetic Activity

Type 2 diabetes impairs insulin synthesis by the pancreas, thereby leading to hyperglycemia. The absorption of simple sugars should be controlled by the inhibitors of enzymes responsible for sugar hydrolysis in the gastrointestinal tract. In turn, obesity and lipid absorption are controlled by pancreatic lipase inhibitors [37,107,108]. Therefore, the antidiabetic activity of *E. multiflora* fruit parts was measured as the inhibitory activity against α-amylase, α-glucosidase, and pancreatic lipase [37]. The authors of the cited study tested six new biotypes of goumi, which were selected in the Experimental Garden of the University of Warmia and Mazury in Olsztyn (north-eastern Poland). The inhibitory activity against α-amylase and α-glucosidase in the fruit skin and pulp of *E. multiflora* reached 24.6 and 32.3 IC_50_ (mg/mL) on average, respectively, whereas the inhibitory activity against pancreatic lipase was 74.9 IC_50_ (mg/mL) on average, implying that the antidiabetic activity of the fruit skin and pulp was three-fold stronger than the antidiabetic activity of seeds and leaves. The highest inhibition of the tested enzymes was noted for the fruit skin and pulp of biotype Si5 (17.0 and 23.7 mg/mL against α-amylase and α-glucosidase, respectively), whereas obesity was most effectively controlled by the fruit skin and pulp of biotype Si4 (69.0 mg/mL against pancreatic lipase). The antidiabetic activity of *E. multiflora* fruit skin and pulp was similar to that noted for the extract of *E. umbellata* [107]. Lee et al. [13] found that the 50% ethanol extract of *E. multiflora* fruit has potent α-glucosidase inhibitory activity and could be an effective antidiabetic agent. α-glucosidase inhibitors can be used in the treatment of many diseases such as diabetes, cancer, and HIV [13,109,110,111], which has contributed to the increasing popularity of cherry silverberry. In a study by Lachowicz et al. [37], the fruit skin and pulp of *E. multiflora* exhibited the strongest antidiabetic properties because their components migrate to juice during pressing. Cherry silverberry juice can be used to produce a functional powdered additive. Furthermore, sugars can be removed from the juice to enhance its antidiabetic effect.

### 4.4. Anticancer Activity

In developed countries, cancer has emerged as the leading cause of premature death. Therefore, effective cancer prevention strategies are being sought. The results of epidemiologic studies have prompted food manufacturers to incorporate plant raw materials containing anticarcinogenic substances in their products [112]. This group of compounds includes lycopene, whose anticarcinogenic properties are associated with its high antioxidant activity. Cherry silverberry fruit is a valuable source of lycopene, which appears to be the most potent antioxidant among common carotenoids, known for its anticarcinogenic effects [14,15,16,17,18,19]. Studies involving cell lines, animals, and human subjects have shown that dietary lycopene can decrease the risk and growth of prostate cancer, ovarian cancer, cervical cancer, breast cancer, esophageal cancer, liver cancer, gallbladder cancer, brain tumors, and cardiovascular disease [109,110,111,112,113,114,115,116,117], as well as tumors of the upper respiratory tract [118].

Cancer is a disease in which some of the body’s cells grow uncontrollably and spread to other parts of the body. The anticancer activity of *E. multiflora* has been confirmed by experiments, with in vitro as well as in vivo models. The mechanisms underlying tumor-suppressing properties, including the ability to remove ROS, interfere with cell division, and modulate the signal transduction pathway, are being investigated [2].

Lee et al. [12] examined the potential of cherry silverberry as a cancer-preventive agent through regulating inflammatory signals, including cyclooxygenase-2 (COX-2) and Akt. Extracts from the seeds and flesh of *E. multiflora* berries were obtained, and COX-2 and Akt activities were analyzed in cherry-silverberry-extract-treated HT-29 colon cancer cells. The study revealed that the analyzed seed extracts reduced cell viability at concentrations above 1600 mg/mL, and, effectively, reduced COX-2 and p-Akt expression. Both seed and flesh extracts inhibited cell growth and induced apoptosis in HT-29 cells. Lee et al. [30] confirmed that cherry silverberry extracts effectively scavenged 1,1-diphenyl-2-picrylhydrazyl (DPPH) radical in vitro, reduced nitric oxide production in LPS-treated macrophages, and inhibited cell proliferation in MCF7, Hela, and SNU-639 cancer cells. According to Lee et al. [12], further research is needed to elucidate the exact molecular mechanism, by which *E. multiflora* fruit induced apoptosis in colon cancer cells.

Several epidemiological studies [15] have suggested the presence of a positive correlation between inflammation and cancer, in particular a strong association between inflammatory bowel disease and a higher incidence rate of colon cancer. Oh and Lee [32] demonstrated that cherry silverberry seeds, in contrast to its flesh, are believed to exert a possible anticancer effect. *Elaeagnus multiflora* seeds are considered to be a candidate for an anticancer functional food in preventive nutrition programs.

Lizardo et al. [81] explored the possibility of adding value to an underutilized fruit, cherry silverberry, through the process of fermentation, which makes it a potential source of functional food and an ingredient for the prevention of colorectal cancer.

## 5. Conclusions

Similar to seabuckthorn, *E. multiflora* has many potential applications in human nutrition, food technology as an ingredient of functional food, cosmetics (including skin cosmetics), and pharmaceuticals as a component of nutraceuticals, medicine, manufacture, and animal nutrition. Cherry silverberry is a promising fruit plant, which perfectly matches the current trends in horticulture by promoting the cultivation of plants with edible fruit that is attractive to both consumers and food producers, on account of its high nutritional value, medicinal properties, and biological activity. Plant species that can be grown without chemicals and constitute rich sources of bioactive substances have attracted considerable interest from researchers worldwide. The identified bioactive compounds can be used to design new functional foods with specific properties. They are found not only in the fruit but also in other plant parts such as the bark, leaves, flowers, and seeds. The seeds are considered to be a candidate for an anticancer functional food in preventive nutrition programs. Nowadays, a healthy lifestyle is gaining increasing popularity, therefore, the health-promoting potential of plants should be further explored.

## Figures and Tables

**Table 1:** The basic chemical composition of cherry silverberry fruit.

Components	Contents	Ref.	Components	Contents	Ref.
Dry weight [%]	12.64–15.55	[9,44]	Amino acids [mg/100 g FW]	89.68	[44]
Total saccharides [%]	5.34–6.30	[9]	serine	13.93	[44]
Monosaccharides [%]	1.54–1.96	[9]	phosphoethanolamine	13.93	[44]
Total free sugars [mg/100 g FW *]	781.44	[44]	alanine	13.16	[44]
fructose	370.34	[44]	β-alanine	13.16	[44]
glucose	401.96	[44]	aspartic acid	4.62	[44]
sucrose	5.80	[44]	phosphoserine	4.62	[44]
trehalose	3.34	[44]	cystine	4.45	[44]
Crude protein [%]	1.29	[44]	methionine	3.89	[44]
Soluble protein [g/100 g FW]	0.48	[44]	phenylalanine	2.85	[44]
pH	3.29	[44]	threonine	2.63	[44]
Crude ash [%]	0.46–0.62	[2,44]	taurine	2.63	[44]
Biominerals [mg/100 g FW]	1353.70–1855.94	[17,44]	tyrosine	2.17	[44]
potassium	1627.44	[44]	leucine	1.41	[44]
magnesium	140.28	[44]	isoleucine	1.16	[44]
sodium	56.70	[44]	valine	1.12	[44]
calcium	14.70	[44]	β-aminoisobutyric acid	1.12	[44]
iron	7.98	[44]	α-aminoisobutyric acid	0.62	[44]
manganese	5.53	[44]	ornithine	0.57	[44]
zinc	2.89	[44]	glutamic acid	0.51	[44]
copper	0.10	[44]	sarcosine	0.51	[44]
lithium	0.20	[44]	Polyphenolic compounds [mg/100 g DW]	417.02–1268.90	[2,8,37]
nickel	0.12	[44]	phenolic acids	1.22–3.80	[2,8,37]
Lipids [g/100 g]	1.40	[1,9]	flavonols	37.29–56.25	[2,8,37]
unsaturated fatty acids account [%], of which	48.70–54.50	[1,9]	hydrolyzable tannins	3.07–10.60	[2,8,37]
α-linolenic acid [%]	17.50–20.80	[1,9]	stilbenes	0.91–1.71	[2,8,37]
linolinic acid [%]	21.80–25.90	[1,9]	polymeric procyanidins	861.36–1197.34	[2,8,37]
oleic acid [%]	19.30–22.70	[9]	Carotenoids [mg/100 g DW]	40.09–170.00	[2,8,37]
Organic acids [g/100 g DW **], of which	18.48–34.11	[2,36]	phytoene	0.93–0.97	[2,8,37]
malic acid account [%]	55–60	[2]	lycopene	39.16–169.00	[2,8,37]
quinic account [%]	11–15	[2]	β-carotene	0.21–0.31	[2,8,37]
tartaric acid account [%]	9–18	[2]	Tocopherols [mg/100 g DW]	2.00–9.93	[37]
Vitamin C [mg/100 g]	4.22–562.72	[9,44]	Chlorophylls [mg/100 g DW]	393.00	[2,37]

* FW, fresh weight; ** DW dry weight.

## Data Availability

MDPI Research Data Policies.

## Sample Availability

Samples of the compounds are available from the authors.

## References

[1] Piłat B., Zadernowski R. (2017). Bioactive Substances—Positive and Negative Effects of their Addition to Foodstuffs. Przemysł Spożywczy.

[2] Lachowicz S., Bieniek A., Gil Z., Bielska N., Markuszewski B. (2019). Phytochemical parameters and antioxidant activity of new cherry silverberry biotypes (*Elaeagnus multiflora* Thunb.). Eur. Food Res. Technol..

[3] Bieniek A., Dragańska E., Prancketis V. (2016). Assessment of climatic conditions for *Actinidia arguta* cultivation in north-eastern Poland. Zemdirb. Agric..

[4] Latocha P. (2017). The Nutritional and Health Benefits of Kiwiberry (*Actinidia arguta*)—A Review. Plant Foods Hum. Nutr..

[5] Czaplicki S., Ogrodowska D., Zadernowski R., Konopka I. (2017). Effect of sea-buckthorn (*Hippophaë rhamnoides* L.) pulp oil consumption on fatty acids and vitamin A and E accumulation in adipose tissue and liver of rats. Plant Foods Hum. Nutr..

[6] Viapiana A., Wesolowski M. (2017). The phenolic contents and antioxidant activities of infusions of *Sambucus nigra* L.. Plant Foods Hum. Nutr..

[7] Bélanger J.J., Pilling D., FAO (2019). The State of the World’s Biodiversity for Food and Agriculture.

[8] Lachowicz S., Kapusta I., Świeca M., Stinco C.M., Meléndez-Martínez A.J., Bieniek A. (2020). In vitro Antioxidant and Antidiabetic potency of fruits and leaves of *Elaeagnus multiflora* Thunb. and their isoprenoids and polyphenolics profile. Antioxidants.

[9] Bieniek A., Piłat B., Szałkiewicz M., Markuszewski B., Gojło E. (2017). Evaluation of yield, morphology and quality of (*Elaeagnus multiflora* Thunb.) biotypes under conditions of north-eastern Poland. Pol. J. Nat. Sci..

[10] Wani T.A., Wani S.M., Ahmad M., Ahmad M., Ganil A., Masoodi F.A. (2016). Bioacrive profile, health benefits and safety evaluation of sea buckthorn (*Hippophaë rhamnoides* L.): A review. Cogent Food Agric..

[11] Bieniek A., Kawecki Z., Piotrowicz-Cieślak A.I. (2002). The content of some organic ingredients in the fruit of less known fruit plants. Biul. Nauk..

[12] Lee M.S., Lee Y.K., Park O.J. (2010). Cherry silverberry (*Elaeagnus multiflora*) extracts exere anti-inflammatory effects by inhibiting COX-2 and Akt signals in HT-29 colon cancer cells. Food Sci Biotechnol..

[13] Lee J.H., Seo W.T., Cho K.M. (2011). Determination of phytochemical contents and biological activities from the fruits of *Elaeagnus multiflora*. Int. J. Food Sci. Nutr..

[14] Nowak K.W., Mielnik P., Sięda M., Staniszewska I., Bieniek A. (2021). The effect of ultrasound treatment on the extraction of lycopene and β-carotene from cherry silverberry fruits. AIMS Agric. Food.

[15] Przybylska S. (2020). Lycopene-a bioactive carotenoid offering multiple health benefits: A review. Int. J. Food Sci. Technol..

[16] Di Mascio P., Kaiser S., Sies H. (1989). Lycopene as the most efficient biological carotenoid singlet oxygen quencher. Arch. Biochem. Biophys..

[17] Yoon K.Y., Hong J.Y., Shin S.R. (2007). Analysis on the Components of the *Elaeagnus multiflora* Thunb. Leaves. Korean J. Food Preserv..

[18] Stahl W., Sies H. (2003). Antioxidant activity of carotenoids. Mol. Aspects. Med..

[19] Patel S. (2015). Plant genus Elaeagnus: Underutilized lycopene and linoleic acid reserve with permaculture potential. Fruits.

[20] Ahmadiani A., Hosseiny J., Semnanian S., Javan M., Saeedi F., Kamalinejad M., Saremi S. (2000). Antinociceptive and antiflammatory effects of *Elaeagnus angustifolia* fruit extract. J. Ethnopharmacol..

[21] Szałkiewicz M., Kawecki Z. (2003). Oliwnik wielokwiatowy (*Elaeagnus multiflora* Thunb.)—Nowa roślina sadownicza. Biul. Nauk..

[22] Grygorieva O., Klymenko S., Ilinska A., Brindza J. (2018). Variation of fruits morphometric parameters of *Elaeagnus multiflora* Thunb., germplasm collection. Potravin. Slovak J. Food Sci..

[23] You Y.H., Kim K.B., An Ch S., Kim J.H., Song S.D. (1994). Geographical Distribution and Soil Characteristics of Elaeagnus Plants in Korea. Korean J. Ecol..

[24] Sakamura F., Suga T. (1987). Changes in chemical components of ripening oleaster fruits. Phytochemistry.

[25] Ismail M., Hussain M., Mahar S., Iqbal S. (2015). Investigation on Total Phenolic Contents of *Elaeagnus Multiflora*. Asian J. Chemstry.

[26] Bieniek A., Lachowicz S. Oliwnik wielokwiatowy—Alternatywa dla produkcji ekologicznej. Proceedings of the Conference materials X FairFruit and Vegetable Industry of TSW, Warsaw Expo.

[27] Shin S.R., Hong J.Y., Yoon K.Y. (2008). Antioxidant properties and total phenolic contents of cherry Elaeagnus (*Elaeagnus multiflora* Thunb.) leaf extracts. Food Sci. Biotechnol..

[28] Kim S.A., Oh S.I., Lee M.S. (2007). Antioxidative and cytotoxic effects of solvent fractions from *Elaeagnus multiflora*. Korean J. Food Nutr..

[29] Kim S.T., Kim S.W., Ha J., Gal S.W. (2014). *Elaeagnus multiflora* fruit extract inhibits melanin biosynthesis via regulation of tyrosinase gene on translational level. Res. J. Biotechnol..

[30] Lee Y.S., Chang Z.Q., Oh B.C., Park S.C., Shin S.R., Kim N.W. (2007). Antioxidant activity, anti-inflammatory activity, and whitening effects of extracts of *Elaeagnus multiflora* Thunb. J. Med. Food.

[31] Bieniek A. (2021). Oliwnik szansa na zwiększenie bioróżnorodności w sadownictwie. Truskawka Malina Jagody.

[32] Kim S., OH S., Lee M. (2008). Antioxidative and Cytoxic Effects of Ethanol Extracts from *Elaeagnus multiflora*. Korean J. Food Nutr..

[33] Houng J.Y., Nam H.S., Lee Y.S., Yoon K.Y., Kim N.W., Shin S.R. (2006). Study on the antioxidant activity of extracts from the fruit of *Elaeagnus multiflora* Thunb. Korean J. Food Preserv..

[34] Chang Z.Q., Park S.C., Oh B.C., Lee Y.S., Shin S.R., Kim N. (2006). Antiplatet aggregation and antiinflammatory activity for extracts of *Elaeagnus multiflora*. Korean J. Med. Crop Sci..

[35] Bieniek A. (2016). „Cud—Jagoda, czyli oliwnik wielokwiatowy. Szkółkarstwo.

[36] Lachowicz S., Bieniek A., Wiśniewski R., Gil Z., Bielska N., Markuszewski B. (2019). Profil parametrów fitochemicznych i właściwości przeciwoksydacyjne owoców oliwnika wielokwiatowego (Elaeagnusmultiflora Thunb.). Materiały z konf. Naukowej “Miejsce ogrodnictwa we wpółczesnym życiu człowieka I ochronie środowiska Warszawa.

[37] Lachowicz-Wiśniewska S., Kapusta I., Stinco C.M., Meléndez-Martínez A.J., Bieniek A., Ochmian I., Gil Z. (2021). Distribution of Polyphenolic and Isoprenoid Compounds and Biological Activity Differences between in the Fruit Skin+ Pulp, Seeds, and Leaves of New Biotypes of *Elaeagnusmultiflora* Thunb. Antioxidants.

[38] Chinnici F., Spinabelli U., Riponi C., Amati A. (2005). Optimization of the determination of organic acids and sugars in fruit juices by ion-exclusion liquid chromatography. J. Food Compos. Anal..

[39] Zielińska A., Nowak I. (2014). Tokoferole i tokotrienole jako witamina E. Chemik.

[40] Hryniewski T. (2008). Drzewa i krzewy. Vademecum Miłośnika Przyrody.

[41] Kołbasina E. (2003). Jagodnyje Liany i Redkije Kustarniki.

[42] Kozioł A. (2020). Anti-aging active substances and application methods based on nanotechnology. Kosmetologia Estetyczna.

[43] Pawlowski R. (2013). Substancje czynne w ziołach. Hod. Trzody Chlewnej.

[44] Kim N.W., Yoo E.Y., Kim S.L. (2003). Analysis on the Components of the Emit of *Elaeagnus multiflora* Thumb. Korean J. Food Preserv..

[45] Mikulic-Petkovsek M., Schmitzer V., Slatnar A., Stampar F., Veberic R. (2012). Composition of sugars, organic acids, and total phenolics in 25 wild or cultivated berry species. J. Food Sci..

[46] Hong J.Y., Cha H.S., Shin S.R., Jeong Y.J., Youn K.S., Kim M.H., Kim N.W. (2007). Optimization of manufacturing condition and physicochemical properties for mixing beverage added extract of *Elaeagnus multiflora* Thunb. fruits. Korean J. Food Preserv..

[47] Mikulic-Petkovsek M.M., Stampar F., Veberic R. (2007). Parameters of inner quality of the apple scab resistant and susceptible apple cultivars (*Malus domestica* Borkh.). Sci. Hortic..

[48] Keutgen A., Pawelzik E. (2007). Modifications of taste-relevant compounds in strawberry fruit under NaCl salinity. Food Chem..

[49] Janda K., Kasprzak M., Wolska J. (2015). Witamina C–budowa, właściwości, funkcje i występowanie. Pom. J. Life Sci..

[50] Yew W.W., Chang K.C., Leung C.C., Chan D.P., Zhang Y. (2018). Vitamin C and Mycobacterium tuberculosis persisters. Antimicrob. Agents Chemother..

[51] Wasiuk E. (2000). Łoch mnogocwietkowyj kak płodowaja kultura. Materiały z VIII Międzynarodowej konferencji sadowniczej pt. Sowremennyje naucznyje issliedowanija w sadowodstwie. Jałta.

[52] Khattak K.F. (2012). Free radical scavenging activity, phytochemical composition and nutrient analysis of *Elaeagnus umbellata* berry. J Medic Plants Res..

[53] Senica M., Stampar F., Mikulic-Petkovsek M. (2018). Blue honeysuckle (*Lonicera cearulea* L. subs. edulis) berry; A rich source of some nutrients and their differences among four different cultivars. Sci. Hortic..

[54] Jarosz M., Rychlik E., Stoś K., Charzewska J. (2020). Normy Żywienia Dla Populacji Polski i ich Zastosowanie.

[55] Bal L.M., Meda V., Naik S.N., Satya S. (2011). Sea buckthorn berries: A potential source of valuable nutrients for nutraceuticals and cosmoceuticals. Food Res. Int..

[56] Glew R.H., Ayaz F.A., Sanz C., VanderJagt D.J., Huang H.S., Chuang L.T., Strnad M. (2003). Changes in sugars, organic acids and amino acids in medlar (*Mespilus germanica* L.) during fruit development and maturation. Food Chem..

[57] Mazza G. (2005). Compositional and functional properties of saskatoon berry and blueberry. Int. J. Fruit Sci..

[58] Zhang Y., Li P., Cheng L. (2010). Developmental changes of carbohydrates, organic acids, amino acids, and phenolic compounds in ‘Honeycrisp’apple flesh. Food Chem..

[59] Olszowy M. (2019). What is responsible for antioxidant properties of polyphenolic compounds from plants?. Plant Physiol. Biochem..

[60] Piłat B. (2014). Owoce rokitnika (*Hippophae rhamnoides* L.) jako źródło substancji biologicznie aktywnych. Ph.D. Thesis.

[61] Teleszko M., Wojdyło A., Rudzinska M., Oszmianski J., Golis T. (2015). Analysis of lipophilic and hydrophilic bioactive compounds content in sea buckthorn (*Hippophae rhamnoides* L.) berries. J. Agric. Food Chem..

[62] Cho K.M., Joo O.S. (2014). Quality and antioxidant charactistics of *Elaeagnus multiflora* wine through the thermal processing of juice. Korean J. Food Preserv..

[63] Lachowicz S., Oszmiański J., Kalisz S. (2018). Effects of various polysaccharide clarification agents and reaction time on content of polyphenolic compound, antioxidant activity, turbidity and colour of chokeberry juice. LWT.

[64] Spínola V., Pinto J., Llorent-Martínez E.J., Castilho P.C. (2019). Changes in the phenolic compositions of Elaeagnus umbellata and Sambucus lanceolata after in vitro gastrointestinal digestion and evaluation of their potential anti-diabetic properties. Food Res. Int..

[65] Meléndez-Martínez A.J. (2019). An overview of carotenoids, apocarotenoids, and vitamin A in agro-food, nutrition, health, and disease. Mol. Nutr. Food Res..

[66] Meléndez-Martínez A.J., Stinco C.M., Mapelli-Brahm P. (2019). Skin carotenoids in public health and nutricosmetics: The emerging roles and applications of the UV radiation-absorbing colourless carotenoids phytoene and phytofluene. Nutrients.

[67] Ignat I., Volf I., Popa V.I. (2011). A critical review of methods for characterisation of polyphenolic compounds in fruits and vegetables. Food Chem..

[68] Delgado-Pelayo R., Hornero-Méndez D. (2012). Identification and quantitative analysis of carotenoids and their esters from sarsaparilla (*Smilax aspera* L.) berries. J. Agric. Food Chem..

[69] Andersson S.C., Olsson M.E., Johansson E., Rumpunen K. (2009). Carotenoids in sea buckthorn (*Hippophae rhamnoides* L.) berries during ripening and use of pheophytin a as a maturity marker. J. Agric. Food Chem..

[70] Eggersdorfer M., Wyss A. (2018). Carotenoids in human nutrition and health. Arch. Biochem. Biophys..

[71] Dias M.G., Olmedilla-Alonso B., Hornero-Méndez D., Mercadante A.Z., Osorio C., Vargas-Murga L., Meléndez-Martínez A.J. (2018). Comprehensive database of carotenoid contents in ibero-american foods. A valuable tool in the context of functional foods and the establishment of recommended intakes of bioactives. J. Agric. Food Chem..

[72] Piłat B., Zadernowski R. (2016). Fruits of sea buckthorn (*Hippophae rhamnoides* L.)—Rich source of biologically active compounds. Postępy Fitoterapii..

[73] Kallio H., Yang B., Peippo P., Tahvonen R., Pan R. (2002). Triacylglycerols, Glycerophospholipids, Tocopherols, and Tocotrienols in Berries and Seeds of Two Subspecies (ssp. sinensis and mongolica) of Sea Buckthorn (*Hippophaë rhamnoides*). J. Agric. Food Chem..

[74] Srinivasan R., Aruna A., Manigandan K., Pugazhendhi A., Kim M., Shivakumar M.S., Natarajan D. (2019). Phytohemical, antioxidant, antimicrobial and antiproliferative potential of *Elaeagnus indica*. Biocatal. Ana Agric. Biotechnol..

[75] Mahomoodally M.F., Zengin G., Aumeeruddy M.Z., Sezgin M., Aktumsek A. (2018). Phytochemical profile and antioxidant properties of two *Brassicaceae* species: *Cardaria draba* subsp. Draba and Descurainia sophia. Biocatalysis Agric. Biotechnol..

[76] Gołba M., Sokół-Łętowska A., Kucharska A.Z. (2020). Health properties and composition of honeysuckle berry *Lonicera caerulea* L. an update on recent studies. Molecules.

[77] Kolniak-Ostek J., Kłopotowska D., Rutkowski K.P., Skorupińska A., Kruczyńska D.E. (2020). Bioactive Compounds and Health-Promoting Properties of Pear (*Pyrus communis* L.) Fruits. Molecules.

[78] Gayer B.A., Avendano E.E., Edelson E., Nirmala A., Johnson E.J., Raman G. (2019). Effects of intake of apples, pears, or their products on cardiometabolic risk factors and clinical outcomes: A systematic review and meta-Analysis. Curr. Dev. Nutr..

[79] Pawlowska E., Szczepanska J., Koskela K., Kaarniranta K., Blasiak J. (2019). Dietary polyphenols in age-related macular degeneration: Protection against oxidative stress and beyond. Oxid. Med. Cell. Longev..

[80] Lizardo R.C.M., Cho H.-D., Won Y.S., Seo K.-I. (2020). Fermentation with mono- and mixedcultures of *Lactobacillus plantarum* and *casei* enhances the phytochemical content and biological activities of cherry silverberry (*Elaeagnus multiflora* Thunb.) fruit. J. Sci. Food Agric..

[81] Lizardo R.C.M., Cho H.-D., Lee J.-H., Won Y.S., Seo K.-I. (2020). Extracts of *Elaeagnus multiflora* Thunb. Fruit fermented by lactic acid bacteria ihibit SW 480 human colon adenocarcinoma via induction of cell cycle arrest and suppression of metastatic potential. J. Food Sci. Health Nutr. Food.

[82] Jung M.-A., Jo A., Shin J., Kang H., Kim Y., Oh D.-R., Choi C.-Y. (2020). Anti-fatigue effects of *Elaeagnus multiflora* fruit extracts in mice. J. Appl. Biol. Chem..

[83] Jung M.-A., Shin J., Jo A., Kang H., Lee G., Oh D.-R., Yun H.J., Im S., Bae D., Kim J. (2020). Alleviating effects of the mixture of *Elaeagnus multiflora* and *Cynanchum wilfordii* extracts on testosteronedeficiency syndrome. J. Appl. Biol. Chem..

[84] Dehghan M.H., Soltani J., Kalantar E., Farnad M., Kamalinejad M., Khodaii Z., Hatami S., Natanzi M.M. (2014). Characterization of an Antimicrobial Extract from *Elaeagnus angustifolia*. Int. J. Enteric. Pathog..

[85] Sá M.B., Ralph M.T., Nascimento D.C.O., Ramos C.S., Barbosa I.M.S., Sá F.B., Lima-Filho J.V. (2014). Phytochemistry and Preliminary Assessment of the Antibacterial Activity of Chloroform Extract of Amburana cearensis (Allemão) AC Sm. against Klebsiella pneumoniae Carbapenemase-Producing Strains. Evid. Based Complementary Altern. Med..

[86] Nageeb A., Al-Tawashi A., Emwas A.M., Al-Talla Z.A., Al-Rifai N. (2013). Comparison of *Artemisia annua* Bioactivities between Traditional Medicine and Chemical Extracts. Curr. Bioact. Compd..

[87] Gurbuz I., Ustun O., Yesilada E., Sezik E., Kutsal O. (2003). Anti-ulcerogenic activity of some plants used as folk remedy in Turkey. J. Ethnopharmacol..

[88] Rawat S., Singh R., Thakur P., Kaur S., Semwal A. (2012). Wound healing Agents from Medicinal Plants: A Review. Asian Pac. J. Trop. Biomed..

[89] Lima-Filho J.V., Martins L.V., de Oliveira Nascimento D.C., Ventura R.F., Batista J.E.C., Silva A.F.B., Taciana Ralpha M., ValençaVaza R., Boa-Viagem Rabello C., da Silvac I.M.M. (2013). Zoonotic potential of multidrug-resistant extraintestinal pathogenic Escherichia coli obtained from healthy poultry carcasses in Salvador, Brazil. Braz. J. Infect. Dis..

[90] Cowann M.M. (1999). Plant products as antimicrobial agents. Clin. Microbiol. Rev..

[91] Uddin G., Rauf A. (2012). Phytochemical screening and biological activity of the aerial parts of *Elaeagnus Umbellate*. Sci. Res. Essays.

[92] Khan S.U., Khan A.U., Ali Shah A.U., Shah S.M., Hussain S., Ayz M., Ayz S. (2013). Heavy metals content, phytochemical composition, antimicrobial and insecticidal evaluation of *Elaeagnus angustifolia*. Toxicol. Ind. Health.

[93] Okmen G., Turkcan O. (2013). The antibacterial activity of *Elaeagnus angustifolia* L. against mastitis pathogens and antioxidant capacity of the leaf methanolic extracts. J. Anim. Vet. Adv..

[94] Okmen G., Turkcan O. (2014). A study on antimicrobial, antioxidant and antimutagenic activities of *Elaeagnus angustifolia* L. leaves. Afr. J. Tradit. Complementary Altern. Med..

[95] Liu J.P., Chang Y.B., Ya-ling J., Xiang Y.G., Chao L. (2001). Study on the Antibacterial Activity of *Elaeagnus Macrophylla* Thunb. Leaf Extract. North. Hortic..

[96] Fenjuan S., Zhiyong C., Ling X., Guiqin Y. (2009). Study on Antimicrobial Activity of the Alkaloids from *Elaeagnus Mollis*. Plant Prot..

[97] Merculieff Z., Ramnath S., Sankoli S.M., Venkataramegowda S., Murthy S.G., Ceballos R.M. (2014). Phytochemical, antioxidant and antibacterial potential of *Elaeagnus kologa* (Schlecht.) leaf. Asian Pac. J. Trop. Biomed..

[98] Arias R.M., Prado A., Hernandez-Perez B.M., Sanchez Mateo C.C. (2002). Antimicrobial studies on three species of *Hypericum* from the Canary Islands. J. Ethnopharmacol..

[99] Mubasher S., Sabir, Dilnawaz S.A., Imtiaz M.H., Kaleem M.T. (2007). Antibacterial activity of *Elaeagnus umbellata* (Thunb.) a medicinal plant from Pakistan. Saudi Med. J..

[100] Lee H.B., Kim C.S., Ahn Y.J. (2004). Anti-helicobacter pylori activity of methanol extracts from Korean native plant species in Jeju Island. Agric. Chem. Biotechnol..

[101] RameshKannan N., Nayagam A.A.J., Gurunagara S., Muthukumar B., Ekambaram N., Manimaran A. (2013). Photochemical screening from *Elaeagnus indica* activity against human pathogens and cancer cells. Adv. Biol. Res..

[102] Nikolaeva G., Krivenchuk P.E. (1971). Prokopenko. Elaeagnus angustifolia flavonoids. Farm..

[103] Zargari A. (1990). Medicinal Plants.

[104] Minhas F.A., Rehaman H., Yasin A., Awan Z.I., Ahmed N. (2013). Antimicrobial activities of the leaves and roots of *Elaeagnus umbellate* Thunb. Afr. J. Biotechnol..

[105] Wang B., Lin L., Ni Q., Lian Su C. (2011). *Hippophae rhamnoides* Linn. For treatment of diabetes mellitus: A review. J. Med. Plants Res..

[106] Lavinia S., Gabi D., Drinceanu D., Stef D., Daniela M., Julean C., Tetileanu R., Corcionivoschi N. (2009). The effect of medicinal plants and plant extracted oils on broiler duodenum morphology and immunological profile. Rom. Biotech. Lett..

[107] Nazir N., Zahoor M., Nisar M., Khan I., Karim N., Abdel-Halim H., Ali A. (2018). Phytochemical analysis and antidiabetic potential of *Elaeagnus umbellate* (Thunb.) in streptozotocin-induced diabetic rats: Pharmacological and computational approach. BMC Complement. Altern. Med..

[108] Saltan F.Z., Okutucu B., Canby H.S., Ozel D. (2017). In vitro α-Glucosidase and α-Amylase Enzyme Inhibitory Effects in *Elaeagnus angustifolia* Leaves Extracts. Eurasian J. Anal. Chem..

[109] Ducep J.B., Kastner P.R., Marshall F.N., Danzin C. (1991). New potent α-glucohydrolase inhibitor MDL 73945 with long duration of action in rats. Diabetes.

[110] Fernandes B., Sagman U., Auger M., Demetrio M., Dennism J.W. (1991). Β-6 branched oligosaccharides as a marker of tumor progression in human breast and clon neoplasia. Cancer Res..

[111] Ogawa S., Maruyama A., Odagiri T., Yuasa H., Hashimoto H. (2001). Synthesis and biological evaluation of α-L-fucosidase inhibitors: 5a-carba- α-L-fucopyranosylamine and related compounds. Eur. J. Org. Chem..

[112] Skiepko N., Chwastowska-Siwiecka I., Kondratowicz J. (2015). Properties of lycopene and utilizing it to produce functional foods. ŻYWOŚĆ Nauka Technol. Jakość.

[113] Bramley P.M. (2000). Is lycopene beneficial to human health?. Phytochemistry.

[114] Larsson S.C., Orsini N., Wolk A. (2006). Processed meat consumption and stomach cancer risk: A metaanalysis. J. Natl. Cancer Inst..

[115] Omoni A.O., Aluko R.E. (2005). The anti-carcinogenic and anti-atherogenic effects of lycopene: A review. Trends Food Sci. Technol..

[116] Rao A.V., Agarwal S. (2000). Role of antioxidant lycopene in cancer and heart disease. J. Am. Coll. Nutr..

[117] Yang T., Yang X., Wang X., Wang Y., Song Z. (2013). The role of tomato products and lycopene in the prevention of gastric cancer: A meta-analysis of epidemiologic studies. Med. Hypotheses.

[118] De Stefani E., Oreggia F., Boffetta P., Deneo-Pellegrini H., Ronco A., Mendilaharsu M. (2000). Tomatoes, tomato-rich foods, lycopene and cancer of the upper aerodigestive tract: A case-control in Uruguay. Oral Oncol..

